# Histological Evidence of Wound Healing Improvement in Rats Treated with Oral Administration of Hydroalcoholic Extract of *Vitis labrusca*

**DOI:** 10.3390/cimb43010028

**Published:** 2021-06-11

**Authors:** Tarsizio S. Santos, Izabella D. D. dos Santos, Rose N. Pereira-Filho, Silvana V. F. Gomes, Isabel B. Lima-Verde, Maria N. Marques, Juliana C. Cardoso, Patricia Severino, Eliana B. Souto, Ricardo L. C. de Albuquerque-Júnior

**Affiliations:** 1 Post-Graduating Program in Health and Environment, Tiradentes University, Av. Murilo Dantas, 300, Aracaju Sergipe 49010-390, Brazil; tbiotec@hotmail.com (T.S.S.); bellinhadorta@hotmail.com (I.D.D.d.S.); svfloresta@hotmail.com (S.V.F.G.); isabel_limaverde@yahoo.com.br (I.B.L.-V.); mnogueiramarques@yahoo.com.br (M.N.M.); juaracaju@yahoo.com.br (J.C.C.); 2 Institute of Technology and Research (ITP), Av. Murilo Dantas, 300, Aracaju 49010-390, Brazil; nelytha@gmail.com (R.N.P.-F.); pattypharma@gmail.com (P.S.); 3 Tiradentes Institute, 150 Mt Vernon St, Dorchester, MA 02125, USA; 4 CEB-Centre of Biological Engineering, University of Minho, Campus de Gualtar, 4710-057 Braga, Portugal; 5 Department of Pharmaceutical Technology, Faculty of Pharmacy, University of Coimbra, Pólo das Ciências da Saúde, Azinhaga de Santa Comba, 3000-548 Coimbra, Portugal

**Keywords:** plant extracts, flavonoids, phenols, oral administration, wound healing, Wistar rats

## Abstract

Plant extracts rich in phenolic compounds have been demonstrated to accelerate wound healing, but their use by oral route has been poorly studied. The leaves of *Vitis labrusca* are rich in phenolic acids and flavonoids. The goal of this study was to assess the healing properties of the oral administration of hydroalcoholic extract of *V. labrusca* leaves (HEVL) in a murine model. HEVL was obtained by Soxhlet and dynamic maceration, and their yield and phenolic acids and flavonoid contents were determined. For the wound healing assay, 8 mm wounds were performed on the back of 48 Wistar rats, assigned into four groups (*n* = 12): CTR (distilled water), HEVL100, HEVL200, and HEVL300 (HEVL at 100, 200, and 300 mg/kg, respectively). On days 7 and 14, wound closure rates were assessed, and the healing wounds were subjected to histological analysis. Soxhlet-obtained extract was selected for the wound healing assay because it provided a higher yield and phenolic acid and flavonoid contents. HEVL significantly reduced leukocytosis in the peripheral blood (*p* < 0.05), accelerated wound closure (*p* < 0.05), and improved collagenization (*p* < 0.05) on day 7, as well as enhanced the epidermal tissue thickness (*p* < 0.001) and elastic fiber deposition on day 14 (*p* < 0.01). Furthermore, HEVL promoted an increase in the histological grading of wound healing on both days 7 and 14 (*p* < 0.01). The doses of 200 and 300 mg/kg provided better results than 100 mg/Kg. Our data provide histological evidence that the oral administration of HEVL improves wound healing in rodents. Therefore, the extract can be a potential oral medicine for healing purposes.

## 1. Introduction

Wound healing is a complex physiological process that occurs to restore skin integrity after injury. This process involves the spatial and temporal synchronization of a variety of cell types with distinct roles in the phases of hemostasis, inflammation, growth, re-epithelialization, and remodeling [1,2]. Chronic wounds are characterized by long-term impaired healing process, in which proper anatomical and functional results are not achieved within an appropriate period of time, usually more than three months [3,4]. Such injuries have become more frequent due to the increased incidence of diseases that hinder wound healing, such as diabetes, obesity, and vascular diseases [5].

The traditional treatment of chronic wounds is based on topical wound protection procedures in order to provide a favorable environment for the development of pathophysiological phenomena related to wound healing [6]. However, several factors must be considered to choose the best route of drug administration in chronic wounds: (i) dysfunctional wound vasculature may reduce the bioavailability of drugs topically delivered; (ii) the wound environment is rich in several pro-inflammatory cytokines that can disable drugs; (iii) wound healing processes may be long-lasting, so drugs must be delivered throughout the healing period [7]. Therefore, the long-term administration of drugs, sometimes with distinctive modulatory activities, might be necessary to provide a proper management of chronic wounds [8].

In recent years, plant extract-derived products have been demonstrated to improve wound healing, either by reducing the influx in situ of inflammatory cells, stimulating angiogenesis and fibroblastic proliferation, accelerating reepithelization, or even promoting a combination of these pathophysiological effects [9,10]. Among the advantages of these products, we highlight the presence of several active chemical compounds, easy access and limited side effects. Different pharmacological targets are involved in the healing effects of herbal products, depending on the phytochemical composition of the extract, including negative modulation of inflammatory cytokines release, stimulation of antioxidant enzymes to reduce oxidative stress, and modulation of angiogenesis [11]. Flavonoids are phytochemicals isolated from a wide variety of plants, represented by more than 8000 chemical compounds, with well-recognized antioxidant and anti-inflammatory effects [12,13,14,15,16]. These properties give flavonoid-rich plant extracts an important potential for use in different formulations for wound healing applications [17].

*Vitis labrusca* is a species of grapevines belonging to the Vitis genus in the flowering plant family Vitaceae, which is cultivated in more than 400 ha in a region called Agreste in Northeastern from Brazil [18]. Hydroalcoholic extracts of leaves of *V. labrusca* (HEVL) are rich in flavonoids, such as quercetin, kaempferol, and naringin [19,20]. Naringin has demonstrated wound healing potential via ghd down-regulation of expression of inflammatory (NF-κB, TNF-α, and ILs) and apoptotic factors (pol-γ and Bax), and up-regulation of growth factor expression (VEGF and TGF-β), thus modulating collagen-1 expression to induce angiogenesis [21]. Quercetin has been proven to improve wound healing by modulating αV and β1 integrin expression [22] and suppressing the MAPK pathway [23]. In addition, kaempferol has been demonstrated to increase the levels of hydroxyproline and maximum tensile strength, as well as accelerate reepithelization in skin wounds of diabetic and non-diabetic rats [24]. Hence, the presence of such flavonoids with wound healing properties in HEVL, as well as their modulatory effects on oxidative stress and the inflammatory state that accompanies wound healing progression, strongly suggests healing potential.

The ideal route of administration of any medication is the one that achieves serum concentrations sufficient to produce the desired effect without inducing any serious adverse side effects. In the oral administration, drugs are mostly absorbed in the small intestine to reach the blood stream and are made available to the target tissues. This is an economical, comfortable, painless route, and can promote both local and systemic effects [25]. The efficiency of drug administration by oral route in experimental models of wound healing in rodents has already been demonstrated in previous studies [2]. Therefore, the goal of this study was to evaluate the potential use of hydroalcoholic extract of *V. labrusca* by the oral route for wound healing in a murine model.

## 2. Materials and Methods

### 2.1. Plant Material

The leaves of Vitis labrusca were collected during November 2017 (from Areia Branca, Sergipe, Brazil, 10°45′29″ S 37°18′45″ O) at the end of flowering and before fruiting. After collection, the leaves were dried under shade, ground in a knife mill until a 32-mesh size was obtained, and stored in amber glass under controlled temperature (25 ± 2 °C) and relative humidity (50 ± 5%).

### 2.2. Extraction Process

The extraction process was performed comparing two commonly used methods: Soxhlet and dynamic maceration. In both extractive procedures, the proportion of solute (dry sample, g): solvent (ethanol 70° g.L, 8 mL) used was 1:16 (*w/v*). The plant powder was continuously extracted for 12 h at a maximum temperature of 45 °C (4 cycles, 60 min) in a Soxhlet apparatus [26]. Dynamic maceration was carried out under constant mechanical shaking (Fisatom 713 D mechanical stirrer, Unique Inc., London, UK, 500 rpm) at room temperature for 24 h. The experiments were carried out in triplicate. Subsequently, the solvent was removed. The dry extract was weighed and the yield was calculated and expressed in mg of dry extract/g of raw material [27].

### 2.3. Assessment of Total Phenolic

The total phenolic content was assessed by Folin-Ciocalteu method using gallic acid as standard. A 0.8 mL aliquot of the extract previously dissolved in methanol (1 mg/mL) was added to a mixture of 9.2 mL of distilled water, 2 mL of Folin-Ciocalteu reagent (Sigma-Aldrich, St. Louis, MO, USA) and 8 mL of 15% Na_2_CO_3_. The obtained solution was stirred for 30 s. The absorbance of the samples was measured after 24 h. The calibration curve was obtained using gallic acid standard solution and the absorbance determined at 750 nm by UV–Vis spectrophotometer (Shimadzu UV 1650, Tokyo, Japan) (y = 0.0179x − 0.0394, R = 0.996). The results were expressed as μg of gallic acid equivalent in relation to the extract mass (μg GAE/g of extract) [28]. All analyses were carried out in triplicate.

### 2.4. Assessment of Total Flavonoids Content

The total flavonoid content was performed as previously described [29], using quercetin as standard. The dry extract was dissolved in methanol (1 mg/mL) and an aliquot of 0.4 mL of this solution was added to 0.5 mL of 2% AlCl_3_. After 30 min, the absorbance was measured at 420 nm at room temperature, against a blank sample without aluminum chloride. The total of flavonoids was calculated using the equation from standard curve of quercetin (y = 0.0087x + 0.0192, R = 0.991) and the results were expressed as μg of quercetin equivalent in relation to the extract mass (μg QE/g extract). The yield of phenolic compounds and flavonoids was calculated using Equations (1) and (2).

(1) $$YGAE=\frac{GAE\times Y}{100}$$

where YGAE is the yield of GAE (μg GAE/g raw material), GAE is mass of phenolic acids equivalent to gallic acid (μgEGA/g extract), and Y is extraction yield (g dry extract/100 g raw material).

(2) $$YQE=\frac{QE\times Y}{100}$$

where YQE is the yield of QE (μg QE/g raw material), QE is mass of flavonoids equivalent to quercetin (μg QE/g extract), and Y is extraction yield (g dry extract/100 g raw material).

### 2.5. Wound Healing Activity

The extract with the highest contents of phenolics and flavonoids was chosen for wound healing activity assay. Ethical principles of the CONCEA (National Animal Experiment Control Council) for experiments in animals were applied in this study, which was approved by the Ethical Committee for Animal Experimentation (approval 020491). Forty-eight male Wistar rats (300 ± 20 g) were anesthetized with intraperitoneal administration of xylazine/ketamine (1:1, 5 mg/kg) and a rounded full-thickness wounds were performed on the back of each animal (1 cm under the interscapular area) using an 8 mm biopsy punch. The animals were randomly assigned into four groups (*n* = 18), as shown in Table 1. After 7 and 14 days, six animals of each group were subjected to euthanasia in CO_2_ chamber (EB 248, 400 × 320 × 350 mm, Insight, São Paulo, Brazil) with a continuous flow of 100% carbon dioxide for 5 min. After death certification, the wounded areas were surgically removed, formalin-fixed, and paraffin-embedded according to routine histotechnical procedures. Serial histological sections (5 μm thick) were obtained and further stained by histochemical techniques [30].

### 2.6. Assessment of Wound Closure Rates (WCR)

Immediately after performing the experimental surgical procedures, as well as after each euthanasia time, the animals were positioned on a plastic white platform and the wounds were photographed using a digital camera (Olympus, C5060, São Paulo, Brazil), at a standard distance of 30 cm from the horizontal base. The images were processed using a morphometry software (Image J^®^, National Institute of Health, Bethesda, MD, USA) to assess the wounded areas (at days 0, 7, and 14). Wound closure rates were obtained according to the Equation (3):

(3) $$WCR=\frac{iWA-fWA}{iWA}\times 100$$

where WCR is the wound closure rate, iWA is initial wounded area (day 0), and fWA is final wounded area (day 7 and day 14). A clinical evaluation was also carried out and a descriptive analysis of the macroscopic (gross) features of the wounds was performed according the following criteria: edema, suppuration, and hyperemia [31].

### 2.7. Histological Analysis of Wound Healing

Twelve histological sections (5 µm thick) were obtained from each paraffin-embedded surgical specimen and stained in hematoxylin-eosin (HE). A descriptive analytical study of the inflammatory infiltrate, granulation tissue, primary fibrous scar (dense connective tissue formed as a result of fibrosis of the granulation tissue), and epithelization process was performed and a comparative analysis among groups was carried out. The histological sections were examined by two examiners, who were blinded to the groups during all the histological analysis performed in this study.

### 2.8. Assessment of the Mean Epidermal Thickness

Five histological fields (400 × magnification, 0.04 mm^2^) of the epidermal tissue over the scarred area were photomicrographed from each histological section. The top-down epidermal thickness was measured in five different points (thickest points) using the software Image J^®^ (version 1.44, (National Institute of Health, Bethesda, MD, USA) to obtain the mean thickness of the skin epithelial lining.

### 2.9. Assessment of Collagen and Elastic Fibers Content

The analysis of collagen fibers was carried out in three histological sections of each animal stained in Sirius red and analyzed under polarized light. Collagen fibers were classified into type III or type I according to their birefringence pattern (green and yellow/red, respectively). The morphological features (stretched/wavy, thin/thick, short/long) and architectural arrangement (reticular, parallel, or interlaced) of the fibers were also observed. Another three sections stained in Weigert were used to assess the content of elastic fibers under conventional light microscopy. In both studies, ten histological fields (400 × magnification, 0.025 mm^2^) of each histological section were photomicrographed and the percentage of the area containing collagen/elastic was obtained using the software Image J^®^.

### 2.10. Assessment of Histological Grading of Wound Healing

From each paraffin-embedded sample 12 (twelve) histological sections were obtained, arranged in glass slides (3 sections per slide, totaling a series of three slides per paraffin-embedded sample). One slide from each series was stained in HE, Sirius red, Weigert and toluidine blue, for analysis under conventional light (HE, Weigert and toluidine blue) and polarized light (picrossirius) microscopy. The histological grading of the experimental healing state was assessed in six serial sections of each animal (three of them stained in HE, under conventional light microscopy, and three in Sirius red, under polarized light microscopy). The analytical procedures were performed using a semi-quantitative scoring system based on an ordinal scale considering six histological parameters related to the healing process, as depicted in Table 2. The total healing score in each case was calculated by adding the scores of individual criteria, with lower scores indicating poorer wound healing. Healing status was graded as good (16–19), fair (12–15) and poor (8–11).

### 2.11. Statistical Analysis

Statistical analysis of the data was performed using the software PRISM 7.0. The data were analyzed regarding their normality distribution using the Shapiro-Wilk test. Gaussian data were analyzed by T student test (extraction data), analysis of variance (ANOVA) and Tukey’s multiple comparisons test. Whereas non-Gaussian data were analyzed using a Kruskal–Wallis test and Dunn’s multiple comparisons test. Chi-square test was used to analyze possible associations between data with categorical distribution (contingency analysis). The significance level adopted was 0.05.

## 3. Results and Discussion

### 3.1. Hydroalcoholic Extract of V. labrusca Obtained Using Soxhlet Extraction Process Provides High Content of Phenolic Acids and Flavonoids

Table 2 shows the comparative the results of both extraction procedures considering the combination of yield and content of total phenolic and flavonoid. The Soxhlet extraction (40 °C/12 h) provided extraction yield significantly higher than dynamic maceration (*p* = 0.0014), and contents of total phenolic and flavonoid results presented values statistically similar to the maceration ones (*p* = 0.3375 and *p* = 0.4668). However, the yield values for GAE and QE were higher for the Soxhlet process, since the extract yield results interfered positively in these values.

The conventional extraction methods, such as, dynamic maceration and Soxhlet are able to extract considerable amounts of active substances with a relatively simple apparatus. Dynamic maceration favors the better diffusion of the bioactive substances through the solvent. In addition, the solvent shake minimizes the concentration of the actives on the sample surface, providing an increase of the rate mass transport compared to static methods, such as Soxhlet extraction. The yield of extraction was better in Soxhlet process, even using a lower time of extraction. Despite both methodologies presenting similar amounts for active substances (phenolic acids and flavonoids), the yield was higher for Soxhlet extraction. The room temperature and the saturation of solvent can be considered limitations for dynamic maceration in this case. The temperature improved the solubilization of the bioactive substances increasing the extraction rate. However, the increase of temperature can degrade or volatilize some important substances. The use of 45 °C for Soxhlet extraction avoided the degradation and volatilization of bioactive substances. Soxhlet extraction is one of the most commonly used methods to obtain bioactive compounds, including in comparison with advanced methods. The second limitation of maceration is the solvent saturation. The renewable process of solvent in Soxhlet process favors the extraction rate and avoid the solvent saturation using the same volume. Thus, the Soxhlet-obtained extract of *V. Labrusca* was selected to be used in the wound healing activity assay.

### 3.2. Hydroalcoholic Extract of V. labrusca Reduces Leukocytosis Counts in Peripheral Blood in Early Stages of Wound Healing

**Figure 1:**
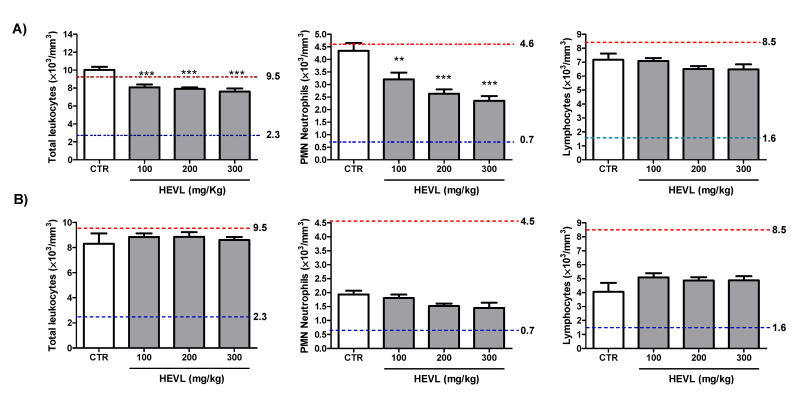
Assessment of leukocyte counts on the peripheral blood of the animals of the experimental groups on day 7 (**A**) and on day 14 (**B**). Red and blue pointed lines represent the upper and lower limits of the reference values. Data are expressed as mean ± standard error mean. Significant differences in comparison with CTR are expressed as ** *p* < 0.01 and *** *p* < 0.001(ANOVA and Tukey’s multiple comparison test).

Total leukocytes, neutrophils, lymphocytes and monocytes counts of the peripheral blood in the experimental groups on days 7 and 14 are shown in Figure 1. Only a mild increase in the total leukocytes count of the peripheral blood was observed in group CTR (10.0 ± 0.3 × 10^3^/mm^3^) over the reference values range on day 7. Oral administration of HEVL not only significantly reduced total leukocyte counts (*p* < 0.001), but also brought those mean counts into the reference range, regardless of the dose. As demonstrated in the differential counts, although all the leukocytes’ counts were within the reference ranges, neutrophils were significantly higher in CTR than in HEVL-treated groups (*p* < 0.05), whereas no significant difference was observed in the lymphocytes and monocytes counts between groups (*p* > 0.05). On day 14, all the leukocyte values were within the normal reference range and no significant difference was observed between groups (*p* > 0.05).

Mild increase in the counts of total leukocytes on day 7 was already expected, as the peak of the inflammatory phase of wound healing has already been gone at this time. The relatively increased counts of neutrophils suggest that the mild leukocytosis observed in CTR is likely because of a residual neutrophilia. Besides, the total leukocytes count reduction induced by HEVL oral administration might be related with the potential anti-inflammatory effects of the high contents of flavonoids present in the extract. Flavonoids can inhibit regulatory enzymes (e.g., cyclooxygenase, protein kinases and phosphodiesterases), promoting decreased release of proinflammatory mediators such as prostaglandins, decreased signal transduction, and decreased cell activation, as well modulate transcription factors (e.g., NF-κB, GATA-3 and STAT-6), which leads to decreased transcription of proinflammatory genes. Those biological activities might have shortened the time-course of the acute phase of inflammation, accelerating the restoration of the normal values of leukocytes counts. On the other hand, on day 14, the remodeling phase of wound healing was already achieved, and no substantial inflammatory response was supposed to be on course in any group [32], which can explain all the white blood cells counts being within the normal reference range, as observed in the current study.

### 3.3. Hydroalcoholic Extract of V. labrusca Accelerates Wound Close in Early Stages of Wound Healing

**Figure 2:**
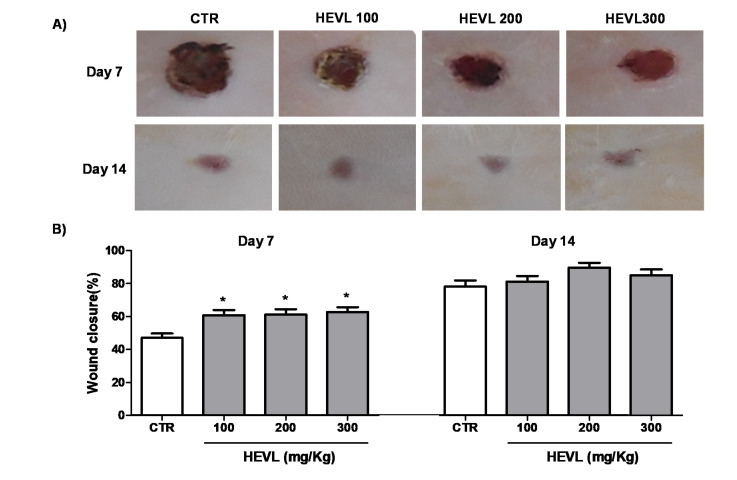
(**A**) Photography of clinical/gross features of the wounds on day 7 and day 14 of the experiment. (**B**) Assessment of the average percentage wound closure in experimental groups on day 7 in comparison to day 1. Data are expressed as mean ± standard error of the mean. Significant differences in relation to the CTR (control) group are expressed as * *p* < 0.05 (ANOVA and Bonferroni’s multiple comparison test).

No clinical or gross sign of intercurrences in wound healing, such as edema, erythema or suppuration in the wound area, was observed over the time-course of the experimental (Figure 2A). These data suggest absence of pathophysiological abnormalities that could modify the natural course of wound healing and suggest that the experimental conditions tested were proper for studying the course of the scar repair [18]. Since the 1980s, the progressive wound closure rates (WCR) represent one of the major clinical signs of scar repair success. Thus, WCR is one of the most important parameters in studies of wound healing in in vivo experimental models. On day 7, the mean values obtained in HEVL-treated groups for doses of 100 (60.8 ± 3.1%), 200 (61.2 ± 3.2%) and 300 mg/Kg (62.7 ± 2.9%) were significantly greater than in CTR group (47.1 ± 2.3%) (*p* < 0.05). However, no significant difference was observed between the latter ones (*p* > 0.05). On day 14, no difference in the wound closure percentages was observed between groups (*p* > 0.05) (Figure 2B).

The increase in WCR in the early stages of the repair process suggests that the oral administration of HEVL accelerated the onset of wound closure, with no signs of adverse clinical events, regardless of the dose tested. Supporting our findings, the healing activity of several chemical compounds mainly present in the HEVL, such as kaempferol [24], quercetin [33], luteolin [34], and curcumin [4], has been previously reported. However, the precise pathophysiological mechanisms behind such biological effects remain unclear. Acceleration of wound closure has been related with increased TGF-β (transforming growth factor β)-induced differentiation of fibroblasts into myofibroblasts (cells with contractile phenotype), promoting contraction and consequent reduction of the wound area. However, flavonoids have been commonly reported to inhibit myofibroblast differentiation, particularly those found as the major chemical compounds of HEVL, such as luteolin [35], kaempferol [36] and quercetin [37]. Therefore, the increase of WCR in the early stages of wound healing seems unlikely to be dependent on increased myofibroblast differentiation. Furthermore, the lack of a significant difference in the WCR between groups at day 14 seems to support the theory that no myofibroblast over-differentiation occurred as a result of HEVL oral administration. On the other hand, our previous data on the white blood cells counts in the peripheral blood provided evidence that HEVL presented anti-inflammatory effects. As downregulation of the inflammatory response by plant secondary metabolites has been reported to successfully accelerate wound healing [38], it is possible to suppose that the improved WCR obtained in HEVL-treated groups might have resulted in potential inhibition of the onset and development of inflammatory phase of wound healing. Thus, a reduction of the time-course of the acute inflammatory phase and downregulation of the intensity of the further chronic inflammation would allow the earlier development of the granulation tissues that cover and fill the wounded area and speed up wound closure.

### 3.4. Hydroalcoholic Extract of V. labrusca Improves Histological Granulation Tissue and Primary Fibrous Scar Formation

**Figure 3:**
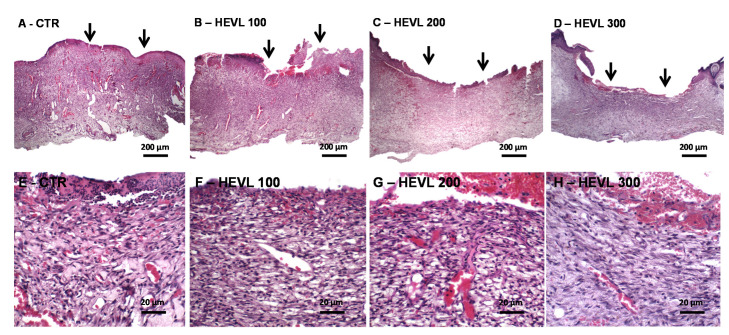
Photomicrographs of histological sections stained in HE representative of the healing wound area in Wistar rats of the experimental in day 7. (**A**–**D**) Panoramic view of CTR and treated with HEVL at doses of 100, 200 and 300 mg/Kg, respectively. Note the non-epithelized wound surfaces (black arrows) (40×). (**E**) CTR shows immature vascular and edematous granulation tissue, whereas (**F**) HEVL 100 and (**G**) 200 presents greater content of proliferative spindle-shape cells (400×). (**H**) In HEVL 300, granulation tissue was less vascular and more cellular (400×).

On day 7, all groups were characterized by granulation, with large strips of granulation tissue involving the full thickness of the dermal tissue, and re-epithelization, which was limited to the marginal areas of the wounds. In CTR group the granulation tissue showed to be immature and rich in hyperemic slip-shaped capillary blood vessels, perpendicularly-arranged in relation to the wound surface. The vascular content network is supported by thin delicate collagen fibrils, in association with moderate spindle-shaped cells proliferation (fibroblasts/myofibroblasts and endothelial cells). Although intense chronic (lympho-histiocytic) inflammatory infiltrate was predominantly observed, persistence of polymorphonuclear infiltrate was noted on the wound surface. Interstitial edema and hemorrhagic areas were common histological findings. The wounds of all the HEVL-treated groups presented more cellular granulation tissue, characterized by spindle and ovoid cells proliferating in long, parallel-arranged fascicles. Chronic inflammatory infiltrate was persistent and ranged from moderate to intense, but the content of collagen fibers filling the intercellular spaces was increased in comparison with CTR. However, edematous changes and inflammation was less intense in HEVL 300 than in the other groups (Figure 3).

The histological features of the healing wounds are consistent with the onset of the proliferative phase of wound healing [39]. During the proliferative phase, growth factors released essentially by macrophages stimulate the proliferation of fibroblasts and endothelial cells (forming new blood vessels), which synthesize, secrete, and anchor in a newly formed extracellular matrix known as granulation tissue.

Neovascularization is critical for efficient wound healing, since it is required for the delivery of nutrients and maintenance of oxygen homeostasis, to allow cellular proliferation and tissue regeneration to occur. New blood vessels formation (angiogenesis) involves activation of local microvascular endothelial cells (ECs), found lining the inner surface of blood vessels. Growth factors, such as VEGF and PDGF, are released in response to a hypoxic wound environment, activating ECs that break down ECM in the granulation tissue, proliferate, migrate, form new cell-cell junctions, and branch out to form new capillaries [40]. Subsequently, fibroblasts promote the progressive deposition of extracellular matrix to form fibrous connective tissue, which gradually replaces the network of newly formed capillaries as it becomes denser and more compact. The gradual maturation of the granulation tissue and subsequent transformation into the primary fibrous scar has been considered one of the important morphological features of wound healing progression [41]. The regulation of the collagen synthesis is controlled by a wide range of growth factors, such as TGF-β1 and FGF, which cause a strong effect upon the genic expression of this protein. Hence, morphological features observed in HEVL-treated groups, consisting of more cellular and fibrous tissue, suggest that the maturation of the granulation tissue is in more advanced stages in comparison with CTR.

**Figure 4:**
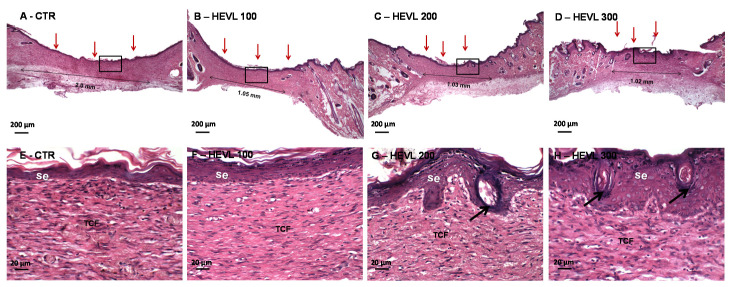
Photomicrographs of HE-stained histological sections representative of the healing wound area in Wistar rats from different groups after 14 days of experiment. (**A**–**D**) Histological panoramic view highlighting the area of scar repair in groups CTR and treated with HEVL at doses of 100, 200 and 300 mg/Kg, respectively. Note the presence of full re-epithelialization of the wound surface (red arrows), as well as the diameter of the CTR healing area considerably larger than in the HEVL-treated groups (thin double-headed arrows) (40×). Higher magnifications show thin keratinized squamous epithelium with no morphological signs of cutaneous appendages differentiation (**E**) CTR and (**F**) HEVL 100 (HE, 400×). (**G**) Group HEVL 200 and (**H**) HEVL 300 exhibiting bulbous buds, most of them with central keratinization, compatible with hair follicles (400×). Black arrows in G and H point at the neoformation of cutaneous appendages (hair follicles). The fibrous connective tissue (TCF) shows persistence of chronic inflammatory infiltrate in CTR whereas inflammation was inconspicuous in the other groups.

On day 14, all HEVL-treated groups presented full re-epithelialized wound surface and, although the reepithelization was quite advanced in CTR, a short non-epithelized area was seen in two cases. CTR exhibited persistence of mature (cellular and considerably less vascular) granulation tissue and mild chronic inflammatory infiltrate, whereas a well-collagenized primary fibrous scar presenting very scanty inflammatory cells and tiny and sparsely distributed blood vessels was observed in HEVL-treated groups. In HEVL 200 and especially in 300, the lining epithelium was shown to be thickened and presented several bulbous buds, sometimes with central keratinization, compatible with rudimentary cutaneous appendages (hair follicles) (Figure 4).

The maturation of the healing tissue observed on day 14 occurs from the decrease in the concentration of hyaluronic acid and the production of interstitial collagens, fibronectin, elastin, and other matrix components by fibroblasts in response to the action of growth factors. During this phase, fibroblasts undergo remarkable phenotypic and functional transformations, initiating the progressive degradation of the high content of collagen, replacing it with fibronectin and proteoglycans. This transformation in the components of the extracellular matrix contributes to cell anchorage and migration, in addition to preventing excessive fibrosis in the formation of the final fibrous scar. We also demonstrated that HEVL oral administration accelerated morphological changes suggestive of improved maturation of the granulation tissue.

Previous studies have demonstrated that, although cultured fibroblasts showed normal growth in response to treatment with quercetin, there was an increase in αV integrin and a decrease in β-1 integrin expression on the cell surface. These changes in the expression of surface integrins appear to be contributing to the stimulation of fibroplasia-related events, including the migration and production of extracellular matrix by fibroblasts. These data could explain the faster fibroblastic maturation of the granulation tissue observed in the groups treated with EHVL. On the other hand, the presence of kaempferol could act as a modulator of this supposedly enhanced fibroblastic action, since this flavonoid is able to inhibit collagen synthesis by fibroblasts in the scar by blocking the receptor for type I TGF-β. As both quercetin and kaempferol have been reported to be major chemical constituents of hydroalcoholic extracts of *V. labrusca* [19,20], the biological activities of these flavonoids could explain the improvement in the granulation tissue maturation and faster transformation into the primary fibrous scar, as well as ultimately prevent the formation of hypertrophic scars at the end of the repair period.

### 3.5. The Highest Doses of Hydroalcoholic Extract of V. labrusca Improves the Thickness of the Squamous Cell Epithelium on Day 14

**Figure 5:**
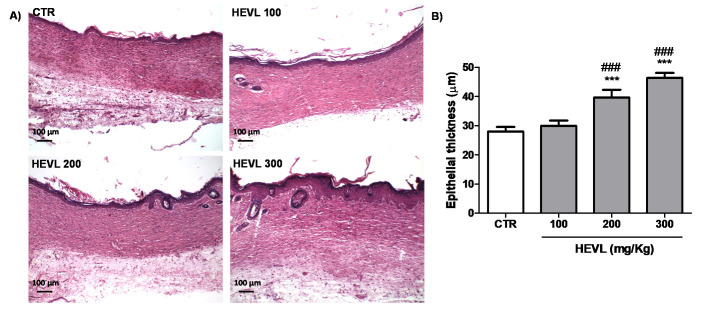
(**A**) Photomicrographs of histological sections stained in HE representative of the scarring tissue in the healing wound area of the experimental in day 14. The scar area is lined by keratinized squamous epithelium with presence of rudimentary hair follicles in HEVL 200 and well-developed hair follicles in HEVL 300 (100×). (**B**) Assessment of the epithelial thickness in the experimental groups on day 14. Data are expressed as median, interquartile range and maximum and minimum values. Significant differences in relation to the CTR (control) group are expressed as *** *p* <0.001; significant differences in comparison with HEVL 100 are expressed as ^###^ *p* < 0.001 (Kruskal-Wallis test and Dunn’s multiple comparison test).

As shown in Figure 5, the epidermal tissue in HEVL treated groups at doses of 200 (39.6 ± 2.6 µm) and 300 mg/kg (46.4 ± 1.7 µm) was significantly thicker than CTR (27.9 ± 1.6 µm) and the group treated with HEVL at 100 mg/kg (29.9 ± 1.8 µm) (*p* < 0.001).

The improvement of the epidermal tissue in the groups treated with HEVL is possibly related to the presence of quercetin in the extract. Quercetin has been previously found to enhance proliferation of human oral keratinocytes via an upregulation of adhesion molecules (Integrin-α6β4) and improved re-epithelization in vitro [42]. Proper keratinocyte proliferation and migration is critical for achieving successful re-epithelialization, which is an essential component of wound healing used as a defining parameter of a successful wound closure. In addition to working as a protective clinical barrier between the environment and the organism, epidermal keratinocytes interact with fibroblasts during wound healing and modulate the expression of matrix metalloproteinases-2 and -9 and their inhibitors, helping to prevent fibrosis. Ultimately, the thicker epithelial line associated with the presence of cutaneous appendages in different developing stages observed in HEVL-treated groups at 200 and 300 might reflect a more mature condition of the epidermal tissue.

### 3.6. Hydroalcoholic Extract of V. labrusca Improves Type III Collagen Deposition in Day 7 of Wound Healing but Do Not Induce Excessive Type I Collagen Production in Day 14

**Figure 6:**
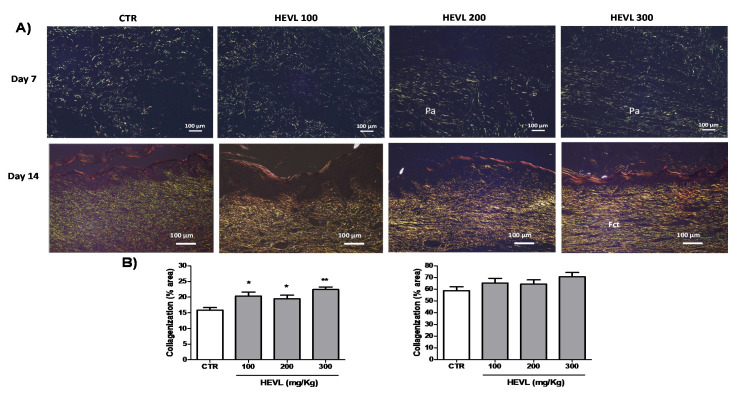
(**A**) Photomicrographs representative of the pattern of collagen deposition in the experimental groups. On day 7, all groups showed deposition of thin and delicate fibrils of type III collagen in a predominantly reticular pattern. On day 14 CTR shows persistence of high content of type III collagen fibers (with green birefringence pattern) interspersed with some delicate type I collagen fibers (with yellow to red birefringence pattern), particularly in the bottom of the wounds. HEVL100 and 200 show predominance of reticular-arranged type I collagen fibers, whereas in HEVL 300 the fibers are thicker and assume a parallel arrangement (Sirius red/polarization, 100×). (**B**) Assessment of collagenization (percentage of collagen fibers content/0.025 mm^2^) within the healing areas in the experimental groups in day 7. Data are expressed as mean ± standard error of the mean. Significant differences in relation to the CTR (control) group are expressed as * *p* < 0.05 and ** *p* <0.01 (ANOVA and Tukey’s multiple comparison test).

In this study, a histochemical analysis of the collagen deposition, based on the birefringence pattern of the fibers, using polarized light microscopy methods was performed to assess the effects of HEVL oral administration on the collagenization over the time-course of wound healing (Figure 6). On day 7, all the groups showed mild deposition of thin and predominantly short collagen fibrils was observed, with reticular arrangement, interspersed by large interfibrillary spaces. Collagen fibrils exhibit a predominantly yellow-green birefringence pattern, consistent with type III collagen. There was no difference in the pattern of collagen deposition between CTR and HEVL 100, but HEVL 200 and 300 showed denser, more regular fibrillary deposition than the other groups. Furthermore, collagen fibrils and fibers assumed reticular disposition in the center and top of the wounds, whereas a parallel-arrangement was evident in the bottom. However, quantitative analysis of the content of collagen fibers revealed that only HEVL 200 (19.1 ± 1.1%; *p* < 0.05) and 300 (22.4 ± 0.8%; *p* < 0.01) presented significantly more intense collagenization than CTR (16.6 ± 0.9%), but no significant difference was observed when CTR was compared with HEVL 100 (20.3 ± 1.2%) (*p* > 0.05). On day 14, a remarkable increase in collagenization, as well as reduction of the interfibrillary spaces, could be observed in all groups. CTR, however, presented the persistence of a substantial content of thin delicate type III collagen (with green birefringence pattern) with variable arrangement, sometimes reticular or parallel. Those fibrils were interspaced with type I collagen fibers, which were more easily found in the bottom of the wounds. HEVL-treated groups showed very similar collagenization pattern, characterized by predominance of thicker and longer type I collagen fibers, homogeneously deposited along the healing area. In addition, type I collagen fibers observed in HEVL-300 appeared to be thicker and present a noticeable parallel-arrangement in relation to the wound surface than in HEVL 100 and 200. However, quantitative analysis of collagenization showed no significant difference in the percentage of collagen fiber contents between CTR (58.7 ± 3.3%), HEVL 100 (65.2 ± 3.9%), HEVL 200 (64.3 ± 3.5%), and HEVL 300 (70.6 ± 3.6%) (*p* > 0.05).

Granulation tissue formation during early stages of wound healing is highly dependent on the production of type III collagen. In days 5–7 of wound healing, fibroblasts migrate into the wound, laying down new collagen of the subtypes I and III. Early in normal wound recovery, type III collagen predominates [43], supporting our morphological findings on day 7. Type III collagen is first synthesized to provide a collagenous scaffold for endothelial cells migration to form the network of capillaries typically seen in the granulation tissue in the early stages of wound healing. In this study, morphological analysis of Sirius-red stained histological sections showed a denser network of type III collagen in HEVL-treated animals at doses of 200 and 300 mg/kg, which was confirmed by histomorphometric analysis. In addition to provide a denser and more regular scaffold for attachment and migration of endothelial cells and developing capillary blood vessels, supporting our previous findings of more rapid formation and maturation of granulation tissue in HEVL-treated groups, higher amounts of type III collagen molecules might have been contributory for wound closure improvement. In fact, impaired wound closure has been previously reported in type III collagen-deficient mice in comparison with controls [44]. This theory seems to support the best rates of wound closure observed in HEVL-treated groups on day 7.

Over the time-course of wound healing the matrix deposited in the wounded area undergoes important changes in its composition. With the closure of the wound, type III collagen is gradually degraded, whereas synthesis of type I collagen increases [45], just as observed in the current study. Type I collagen accumulation speeds up reaching a peak at around day 10–14 when it accounts for at least 75% of total collagen in the granulation tissue [41]. The regulation of the collagen synthesis is controlled by a wide range of growth factors, such as TGF-β1 and FGF, which cause a strong effect upon the genic expression of this protein [46]. Throughout the further remodeling phase, type I collagen molecules undergo enhanced intermolecular crosslinking, which enhances the wound breaking strength, although the original strength of the normal dermis is never fully restored. The persistence of substantial amounts of reticular-arranged type III collagen fibers in CTR in contrast with the clear predominance of parallel-arranged type I collagen fibers in the other groups seems to suggest an improvement of collagen replacement in response to HEVL oral administration. However, no significant difference in the collagenization was observed in the histomorphometric analysis of collagen fiber deposits. Hence, we hypothesized that despite the treatment with flavonoid-rich extract accelerated the replacement of type III for type I molecules, it also modulated the collagenization process, avoiding excessive production of collagen fibers. In fact, quercetin has previously proved to suppress the signaling pathways activating RAW264.7 macrophages and dermal fibroblasts, which is associated with inhibition of multiple tyrosine kinases and simultaneously downregulate the inflammatory and fibrotic responses in injured tissues. Quercetin also increases both mRNA and protein levels of TGF-β3, an anti-fibrotic cytokine, without affecting pro-fibrotic TGF-β1 production [42]. In addition, Kaempferol treatment has demonstrated to significantly reduce mRNA expression levels of inflammatory and pro-fibrotic cytokines, including IL-6, TNF-α, and TGF-β in the lesioned skin. Furthermore, the reduction of the type III Collagen network during granulation tissue formation upregulates myofibroblast differentiation and potentially enhances scar deposition [44]. Thus, the increased deposition of these molecules on day 7, as observed in HEVL-treated groups, might have reduced the risk of further excessive deposition of type I collagen during wound healing, as observed in this study.

### 3.7. The Highest Doses of Hydroalcoholic Extract of V. labrusca Improves Elastic Fibers Deposition on Day 14

**Figure 7:**
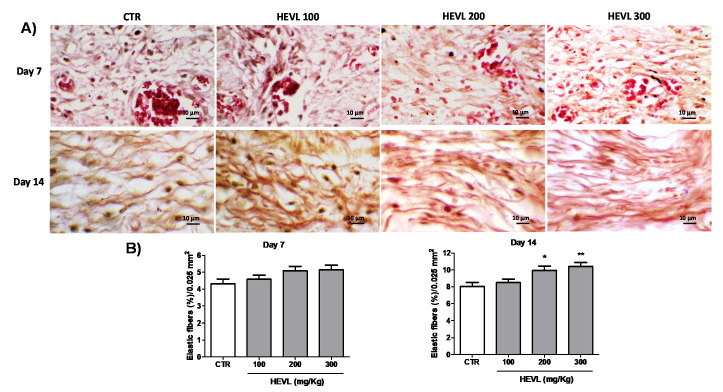
(**A**) Photomicrographs representative of the pattern of elastic fibers distribution in the experimental groups. Elastic fibers are identified as short thin delicate dark-stained fibrillary structures that are scarcely seen on day 7 but more noticeable and irregularly organized on day 14 (Weigert stain, 1000×). (**B**) Assessment of elastic fibers deposition (percentage of collagen fibers content/0.025 mm^2^) within the healing areas in the experimental groups on days 7 day 14. Data are expressed as mean ± standard error of the mean. Significant differences in comparison with CTR are expressed as * *p* < 0.05 and ** *p* < 0.01 (ANOVA and Tukey’s multiple comparisons test).

As demonstrated in Figure 7, elastic fibers were identified as very short thin and delicate dark-stained fibrillary structures, which were disposed in parallel or reticular arrangement, which were associated with grosser and thicker fibers collagen fibers. On day 7, elastic fibers were very scarce within the granulation tissue, and no remarkable difference was observed between groups. This was confirmed by histomorphometric analysis, which showed no significant difference in the elastic fibers content of the experimental groups (*p* > 0.05) On the 14 they were more noticeable, irregularly distributed and interlaced with longer and thicker collagen fibers. In addition, the relative content of elastic fibrils seemed to be more noticeable in HEVL-tread groups at doses of 200 and 300 ng/kg, and this finding was confirmed by the histomorphometric analysis (*p* < 0.01 and *p* < 0.01, respectively).

The elastic fibers are composed of relatively free and unstructured polypeptide chains covalently crossed to form an elastic net that allows the tissues to extend without damage. The interlacement of this fibrillary elastic network with collagen fibers limits the expansion and avoids the laceration of the dermal tissue, playing a key role in the formation and improvement of final appearance of the scar at late stages of wound healing [47]. Furthermore, we provide histological evidence of a beneficial effect HEVL administration on the elastic fibers production during wound healing. As the direct effect of flavonoids, as isolated molecules or as chemical compounds of plant extracts has not been reported so far, the precise mechanisms underlying this biological activity is unclear. The oxidative stress has been proved to induce enhanced cross-linking of the tropoelastin molecules with other proteins required for elastic fiber assembly, including fibulin-4, fibulin-5, and fibrillin-2, making them unable to properly form elastic fibers in an in vitro model (AKHTAR et al., 2010). Therefore, a possible role of the antioxidant activity of the flavonoids present in the HEVL on earlier stages of wound healing might be indirectly associated with the improvement of elastic fibers content on day 14. However, further investigations must be performed to prove this theory right.

### 3.8. Hydroalcoholic Extract of V. labrusca Improves Histological Grading of Wound Healing over the Time Course of the Experimental

As in dermal wound healing assays performed in rodent model pathological features of the granulation tissue and primary fibrous scar have been more frequently assessed on day 7 and 14 days, respectively [27,48], those experimental times were chosen to assess the histological grading of wound healing. A variety of grading systems based on the histological analysis of the healing process, including angiogenesis, inflammation, fibroplasia and restoration of the connective tissue matrix, wound contraction and remodeling, epithelialization, and differentiation, has been proposed to assess the morphological features of wound healing progression [47]. In the current study, the scoring system of six histological parameters described by Sultana et al. (2009) [49], that included the most relevant morphological features that characterize granulation tissue and scar formation/maturation (Table 3), was selected and applied to assess wound healing progression.

**Figure 8:**
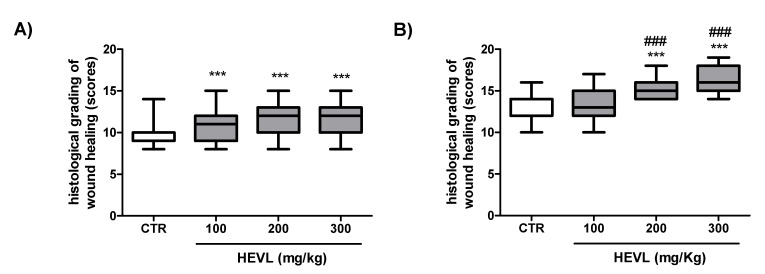
Assessment of wound healing histological grading scores in the experimental groups on day 7 (**A**) and 14 (**B**). Data are expressed as median, interquartile range and maximum and minimum values. Significant differences in relation to the CTR (control) group are expressed as *** *p* <0.001; and significant differences in comparison with HEVL 100 are expressed as ^###^ *p* < 0.001 (Kruskal-Wallis test and Dunn’s multiple comparison test).

On day 7, the scores of histological grading of wound healing, obtained in the CTR group (9; 8–10) were significantly lower than in the HEVL-treated groups 100 (11; 9–12), 200 (12; 10–13) and 300 (11; 10–13) (*p* < 0.001), but no significant difference was observed when the three doses tested were compared with each other ( *p*> 0.05). The profile obtained in day 14 was a little different, as CTR (12; 12–14) and HEVL 100 (13; 12–15) were significantly lower than in HEVL-200 (15; 14–16) and HEVL-300 (18; 16–19) (*p* < 0.001), but no significant difference was observed when the last two groups were compared with each other ( *p*> 0.05) (Figure 8). In addition, morphometric assessment of the epidermal tissue demonstrated that the squamous epithelium in HEVL 200 (39.63 ± 2.6 µm) and 300 (46.92 ± 1.7 µm) was significantly thicker than in CTR (27.99 ± 1.6 µm) and HEVL 100 (29.9 ± 1.8 µm) (*p* < 0.001) (Figure 8).

Histological assessment should include the basic components of the healing process, e.g., angiogenesis, inflammation, fibroplasia and connective tissue matrix restoration, wound contraction and remodeling, epithelialization, and differentiation [50]. The amount of granulation tissue, inflammatory infiltrate, orientation of collagen fibers/fibrils, collagen deposition pattern, immature fibrillar collagen content, and fibrous mature collagen content have been considered the main parameters to analyze the progress of wound healing in experimental models. The comparison of histological patterns with known physiological variations in tissue morphology helps in the qualitative derivation of the diagnosis. The histological improvement of wound healing based on the scoring grading proposed by Sultana et al. (2009) [49] was likely a result of the sum of different beneficial effects induced by the extract that occurred at both early and late stages of the experiment. Interestingly, although wound closure rates were not significantly different between groups, the histological scores were greater in HEVL-treated animals, mainly at higher doses (200 and 300 mg/kg). These data suggest that there is a relevant impact of the oral administration of HEVL on the morphological features of the healing process and justifies the use of the extract by oral route.

## 4. Conclusions

In this study, we analyzed the effect of hydroalcoholic extract of *V. labrusca* on wound healing in a rodent model. Taken together, the results obtained in the current study provide evidence that the administration of the extract by oral route can successfully accelerate a variety of early and late pathophysiological steps that lead to improved wound healing. Based on pathological examinations, hydroalcoholic extract of *V. labrusca* improved wound closure, morphological features of the granulation tissue maturation, and type III collagen formation at early stages of wound healing (day 7). In addition, the extract also improved epidermal thickness, morphological features of the primary fibrous scar, and elastic fiber deposition at late stages (day 14). Thus, the biological effects would be ultimately responsible for improving the histological grading scores of wound healing. Further investigations should be designed in order to evaluate the precise mechanisms underlying healing properties related to the oral administration of hydroalcoholic extract of *V. labrusca* in a rodent model of wound healing.

## Figures and Tables

**Table 1:** Distribution of the animals into the four experimental groups according to the treatment.

Groups (*n* = 12)	Oral Treatment	Dose (Volume)
CTR	Distilled water	–(1 mL)
HEVL 100	Hydroalcohic Extract of *V. labrusca*	100 mg/kg (1 mL)
HEVL 200	Hydroalcohic Extract of *V. labrusca*	200 mg/kg (1 mL)
HEVL 300	Hydroalcohic Extract of *V. labrusca*	300 mg/kg (1 mL)

**Table 2:** Comparative analysis of the extraction methods considering the extraction yield and content of total phenolic and flavonoid.

Extraction Method	T (°C)	Time (h)	Yield (%)	Total Phenolic (μg GAE/g)	Y_GAE_ (μg GAE/g Raw Material)	Total Flavonoid (μg QE/g)	Y_QE_ (μg QE/g Raw Material)
Soxhlet	45	12	27.0 ± 2.4 ^a^	270.1 ± 38.8 ^a^	73.0 ± 13.4 ^a^	78.4 ± 21.5 ^a^	21.5 ± 7.6 ^a^
Maceration	25	24	11.8 ± 2.3 ^b^	232.6 ± 45.3 ^a^	27.8 ± 8.8 ^b^	63.3 ± 24.2 ^a^	7.2 ± 1.8 ^b^
*p* value			0.0014	0.3375	0.0081	0.4668	0.0336

Different letters in the same column mean significantly different values (T student test).

**Table 3:** Histological parameters assessed to calculate f wound healing state.

Histological Parameter	Scoring System	Microscopy (Histochemical Technique)
Amount of granulation tissue	profound-1, moderate-2, scanty-3, absent-4	Conventional light microscopy (HE stained)
Inflammatory infiltrate	plenty-1, moderate-2, a few-4	Conventional light microscopy (HE stained)
Collagen fiber orientation	vertical-1, mixed-2, horizontal-4	Polarized light microscopy (Sirius red stained)
Pattern of collagen	reticular-1, mixed-2, fascicle-4	Polarized light microscopy (Sirius red stained)
Amount of early collagen	profound-1, moderate-2, minimal-3, absent-4	Polarized light microscopy (Sirius red stained)
Amount of mature collagen	profound-1, moderate-2, minimal-4	Polarized light microscopy (Sirius red stained)

## Data Availability

Data are available from corresponding authors upon request.
